# K-shell Analysis Reveals Distinct Functional Parts in an Electron Transfer Network and Its Implications for Extracellular Electron Transfer

**DOI:** 10.3389/fmicb.2016.00530

**Published:** 2016-04-20

**Authors:** Dewu Ding, Ling Li, Chuanjun Shu, Xiao Sun

**Affiliations:** ^1^State Key Laboratory of Bioelectronics, School of Biological Science and Medical Engineering, Southeast UniversityNanjing, China; ^2^Department of Mathematics and Computer Science, Chizhou CollegeChizhou, China

**Keywords:** *c*-type cytochrome, extracellular electron transfer, k-shell decomposition, protein disordered region, protein–protein interaction network

## Abstract

*Shewanella oneidensis* MR-1 is capable of extracellular electron transfer (EET) and hence has attracted considerable attention. The EET pathways mainly consist of *c*-type cytochromes, along with some other proteins involved in electron transfer processes. By whole genome study and protein interactions inquisition, we constructed a large-scale electron transfer network containing 2276 interactions among 454 electron transfer related proteins in *S. oneidensis* MR-1. Using the k-shell decomposition method, we identified and analyzed distinct parts of the electron transfer network. We found that there was a negative correlation between the *k*_s_ (k-shell values) and the average *DR_100* (disordered regions per 100 amino acids) in every shell, which suggested that disordered regions of proteins played an important role during the formation and extension of the electron transfer network. Furthermore, proteins in the top three shells of the network are mainly located in the cytoplasm and inner membrane; these proteins can be responsible for transfer of electrons into the quinone pool in a wide variety of environmental conditions. In most of the other shells, proteins are broadly located throughout the five cellular compartments (cytoplasm, inner membrane, periplasm, outer membrane, and extracellular), which ensures the important EET ability of *S. oneidensis* MR-1. Specifically, the fourth shell was responsible for EET and the *c*-type cytochromes in the remaining shells of the electron transfer network were involved in aiding EET. Taken together, these results show that there are distinct functional parts in the electron transfer network of *S. oneidensis* MR-1, and the EET processes could achieve high efficiency through cooperation through such an electron transfer network.

## Introduction

The transmission of electrons to extracellular solid acceptors (extracellular electron transfer, EET) is an important reaction in some microorganisms, such as *Geobacter sulfurreducens* and *Shewanella oneidensis* (Shi et al., 2007). *C*-type cytochromes play important roles in the EET processes (Shi et al., 2012; Tremblay and Zhang, 2015), for example, four *c*-type cytochromes, CymA, MtrA, MtrC, and OmcA, can form an electron transfer chain with a trans-outer membrane protein MtrB in *S. oneidensis* MR-1 (the classical MtrCAB pathway). Some other EET pathways (such as MtrDEF and the dimethyl sulfoxide (DMSO) pathway) have also been proposed in recent years (Gralnick et al., 2006; Coursolle and Gralnick, 2010, 2012; Breuer et al., 2012, 2014). However, because of the diversity of *c*-type cytochromes, *Shewanella* can express different *c*-type cytochrome genes in different environments. Thus it remains challenging to accurately characterize the EET processes in such species.

Previous studies have revealed the high efficiency of the prediction of biological pathways from biological networks (Planas-Iglesias et al., 2012; Huang et al., 2013; Mukhopadhyay and Maulik, 2014). Following initial work on a small-scale *c*-type cytochrome network (Zhang et al., 2008), a recent study constructed a network for all of 41 *c*-type cytochromes in *S. oneidensis* MR-1 and the classical EET pathways (e.g., MtrCAB, MtrDEF) can be identified from the *c*-type cytochrome network (Ding et al., 2014). Furthermore, from the view of steric properties of individual proteins, Volkov and van Nuland (2012) performed extensive conformational sampling, mapped out functional epitopes in *c*-type cytochrome complexes (involving cytochrome *c* and other redox-active proteins such as peroxidase and cytochrome *b*_5_) and then assessed the electron transfer properties of such interactions.

To take advantage of the extracellular solid electron acceptors which widely exist in cellular living environment, *Shewanella* species develop effective EET strategies based upon *c*-type cytochromes and other redox-active proteins. Here, we explored such processes by constructing an electron transfer network and analyzing its formation and extension, as well as the functional parts in the network. Proteins in the core of a genome-scale protein–protein interaction (PPI) network have a high probability of being essential (Wuchty and Almaas, 2005), and network peripheral proteins tend to be preferentially involved in recent or ongoing adaptive events (Kim et al., 2007), therefore such a core-periphery structure can be helpful to understand PPI networks. Furthermore, k-shell analysis has been widely used to explain both network formation and current structure (Kitsak et al., 2010; Pei et al., 2014), we thus engaged it in this study.

After whole genome study and identification of interactions of proteins that are potentially involved in electron transfer processes in *S. oneidensis* MR-1, a large-scale electron transfer network was constructed (see Construction of the Electron Transfer Network). Then, by integrating protein disordered regions and subcellular localization data, we found that the k-shell structure can be helpful for understanding the formation and extension of the electron transfer network (see K-shell Structure of the Electron Transfer Network). Finally, the functional significance of the various shells in the network is discussed in this paper (see The Top Three Shells Take Charge of Electron Generation, The Fourth Shell Is Responsible for Extracellular Electron Transfer, The *c*-type Cytochromes in the Remaining Shells Are Involved in Aiding Extracellular Electron Transfer).

## Materials and Methods

### Protein Selection

*C*-type cytochromes, which play the most important roles in the EET processes, were identified from genome annotation data (Meyer et al., 2004), and then were verified according to the literature. Other proteins that are potentially involved in electron transfer processes (such as pilin proteins, flavoproteins, and various redox-active proteins) were manually selected from the complete genome of *S. oneidensis* MR-1 (Heidelberg et al., 2002) via the KEGG genome database ^1^.

### Network Construction

Interaction information for these manually selected proteins was obtained from the famous protein interaction database STRING^2^ (Franceschini et al., 2013). Furthermore, experimentally identified and verified interactions from the literature were also considered. Then, the PPI network was built based on these interaction data. GO biological process and KEGG pathway enrichment analyses were carried out using STRING online tools.

### K-shell Analysis

As described elsewhere (Kitsak et al., 2010; Pei et al., 2014), the k-shell decomposition method assigns a *k_s_* value to each node in a network. Such values can be obtained by successive pruning of nodes level by level. That is, removing all nodes with degree *k* = 1 and repeatedly making such procedure, until there are no remaining nodes with degree *k* = 1; all such removed nodes are then assigned a *k_s_* value with *k_s_* = 1. Then, via a similar procedure, one can iteratively obtain the next *k_s_* value (*k_s_* = 2), and so on until all nodes are removed.

### Disordered Regions

Protein disordered regions are functionally versatile and can mediate new interactions of proteins (Buljan et al., 2013; Uhart and Bustos, 2014); they thus play an important role in the formation and extension of PPI networks. The disordered regions of proteins were identified with the tool IsUnstruct (v2.02^3^) (Lobanov and Galzitskaya, 2011). All disordered segments with two or more continuous amino acid residues were considered as disordered regions.

### Subcellular Localization

The subcellular localization of proteins contributes to understand EET processes and the role of different proteins in EET; in this study, it was performed by the following procedures: (1) using PSORTb^4^ (Yu et al., 2010), which is one of the best tools for current subcellular localization analysis; then, (2) using CELLO^5^ (Yu et al., 2006) for proteins that were not resolved by PSORTb; and at last, (3) referring to specific-protein-related literature or known molecular function for checking or revising of the subcellular localization.

### Protein Domains and Their Interactions

Protein (families) domains were mainly determined from Pfam (release 27.0^6^) (Finn et al., 2014), and proteins without domain information in Pfam were analyzed by the prediction tool FFAS^7^ [note that if the predicted templates had overlap area, only the template with the best score was chosen (Jaroszewski et al., 2005)]. Protein domain-domain interactions (DDIs) were mainly resolved from 3did^8^ (Mosca et al., 2014).

## Results And Discussion

### Construction of the Electron Transfer Network

*C*-type cytochromes play important roles in the transmission of electrons from intracellular space to extracellular acceptors (Shi et al., 2012; Tremblay and Zhang, 2015). These highly water soluble proteins covalently bind heme via two cysteine residues. Using pattern matching (the heme-binding CXXCH motif), Meyer et al. (2004) identified 42 genes encoding *c*-type cytochromes in the *S. oneidensis* MR-1 genome. However, according to published literatures, we found that SO_4570 is a pseudo gene and SO_3623 is a degenerate gene; these two genes were thus eliminated. SO_1748 was identified as a periplasmic monoheme *c*-type cytochrome following several recent reports (Romine et al., 2008; Gao et al., 2010; Jin et al., 2013). Furthermore, other proteins that play roles in electron transfer were identified by analyzing genome-wide annotation data. The main types of these proteins are: pilin proteins, flavoproteins, quinone/ubiquinone oxidoreductases, and other various redox-active proteins (e.g., flavodoxins, ferredoxins, and metalloproteases). Overall, 481 proteins were identified (**Supplementary Data Sheet S1**).

Next, we needed to obtain all interaction information on these 481 proteins. Because large-scale experimental data are not presently available, we performed protein interaction inquisition in STRING. The most important parameter for protein interaction inquisition in STRING is the confidence score, which is defined as the approximate probability that a predicted interaction exists between two proteins in the same metabolic map in the KEGG database. To obtain more comprehensive information, medium confidence (0.4) was set. The interaction information was then obtained (**Supplementary Data Sheet S2**, December 2014) and a large-scale electron transfer network was constructed accordingly. After removing isolated nodes (23 proteins) and separated links (two interactions), we obtained a network with 2266 interactions among 454 proteins (**Supplementary Methods**). Furthermore, experimentally identified and verified interactions were also considered. We found that most of the experimentally verified interactions were included in STRING’s predictions. Nevertheless, 10 further interactions were discovered by literature retrieval (Borloo et al., 2011; Schutz et al., 2011; Fonseca et al., 2013) (**Supplementary Methods**). Overall, 2276 interactions among 454 proteins were determined in the final electron transfer network.

The electron transfer network was then modeled as an undirected graph, in which nodes represent the proteins and the links represent protein interactions. It is clear that network structure strongly correlates with its function, for example, several classical EET pathways have been identified by modular analysis of a *c*-type cytochrome network (Ding et al., 2014). As k-shell analysis has been widely used to explain both the formation and current structure of networks (Kitsak et al., 2010; Pei et al., 2014), we thus engaged this method to study the formation and extension of *Shewanella* electron transfer network and its functional parts, as well as their potential implications for EET processes.

### K-shell Structure of the Electron Transfer Network

**FIGURE 1:**
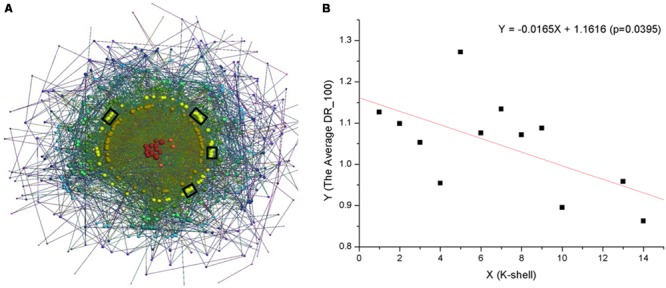
**(A)** Visualization of the k-shell structure of the *S. oneidensis* MR-1 electron transfer network, using the program LaNet-vi (Beiro et al., 2008). The nodes are ordered by their *k*_s_ values. Black panes indicate proteins that form representative EET pathways. **(B)** The negative correlation between the *k*_s_ and the average *DR_100*.

We first performed k-shell decomposition for the *S. oneidensis* MR-1 electron transfer network (**Figure 1A**, **Supplementary Methods, Supplementary Data Sheet S3**). From the network formation and extension view, it is clear that the nodes with high k-shell values (*k*_s_) were those connected initially (Kitsak et al., 2010; Pei et al., 2014). These nodes form a core network that was reconstructed and expanded as new nodes were constantly connected. Since PPIs are strongly influenced by their local environment, the protein interaction system is constantly reconstructed (e.g., by rewiring interactions among existing proteins [Kim et al., 2012]) and expanded (e.g., by recruiting new proteins into the network for highly specific and/or more efficient functions [Nam et al., 2012]) according to changes in the environment. As a result, the structure of the PPI network evolves. Therefore, such a k-shell network structure reflects the formation and extension of the PPI network.

To further explore the formation and extension of the electron transfer network, we considered an important feature of proteins, namely disordered regions. Such disordered regions cannot fold into stable three dimensional structures but increase the functional versatility of proteins; they can also mediate new protein interactions (Buljan et al., 2013; Uhart and Bustos, 2014) and thus play an important role in the formation and extension of PPI networks. For each protein, we first obtained its disordered regions (**Supplementary Data Sheet S4**) and computed the disordered regions per 100 amino acids (*DR_100*). Since many disordered regions locate at the termini of proteins, small proteins will significantly perturb the statistical results. Therefore, the proteins that are less than 100 amino acids were excluded (19 proteins). Then, we analyzed the average *DR_100* for the proteins in every shell of the electron transfer network, and found that there was a negative correlation between the *k*_s_ and the average *DR_100* (*DR_100*_Ave_ = -0.0165 ×*k*_s_ + 1.1616, *p* = 0.0395) (**Figure 1B**).

The results demonstrated that there has been a selection preference for proteins during the formation and extension of the electron transfer network. Proteins with fewer disordered regions seem to have been preferably selected in the core of the electron transfer network. Because the intrinsically disordered regions in proteins do not fold into stable structures under physiological conditions, the proteins in the core of the electron transfer network, with less disordered regions, have a high probability of being stable. The stability of these proteins could favor formation of the macromolecular complexes required to carry out essential cellular processes. For example, metabolic proteins have been shown to possess the lowest disordered content (Pavlovic-Lazetic et al., 2011). In contrast, with the more disordered regions in network peripheral proteins suggests there are frequent dynamic interactions, since protein disordered regions are functionally versatile, and allow the same polypeptide to undertake different interactions with different consequences (Wright and Dyson, 2015). Protein disordered regions can interact with numerous different partners by using molecular recognition features (or linear motifs), participate in the assembly of protein complexes, and provide accessible sites for post-translational modification (van der Lee et al., 2014; Wright and Dyson, 2015), which enables that proteins in the network periphery can aid essential cellular processes and/or function in a wide variety of environmental conditions.

**FIGURE 2:**
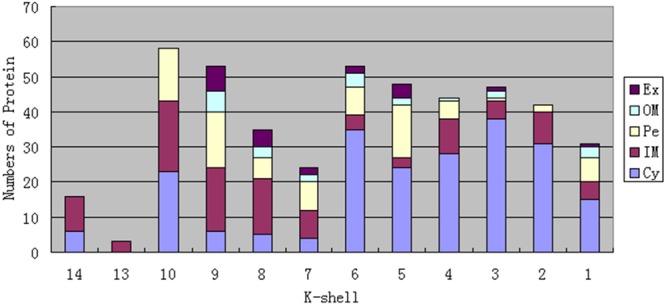
**Numbers of proteins and their subcellular localization in every shell in the *S. oneidensis* MR-1 electron transfer network.** Ex, Extracellular; OM, Outer Membrane; Pe, Periplasm; IM, Inner Membrane; Cy, Cytoplasm.

As previous studies have indicated that nodes with similar *k*_s_ values in a network have equal importance (Kitsak et al., 2010; Pei et al., 2014), and proteins with similar interactions (or topology) in PPI networks have been widely recognized to carry out similar functions (Vazquez et al., 2003; Radivojac et al., 2013; Davis et al., 2015). We thus speculated that various shells in the network might take different biological functions. To address this point, we first analyzed proteins with different *k*_s_ values and their subcellular localization (**Figure 2**, **Supplementary Data Sheet S5**). We discuss the biological significance of the different shells in the following sections.

### The Top Three Shells Take Charge of Electron Generation

**FIGURE 3:**
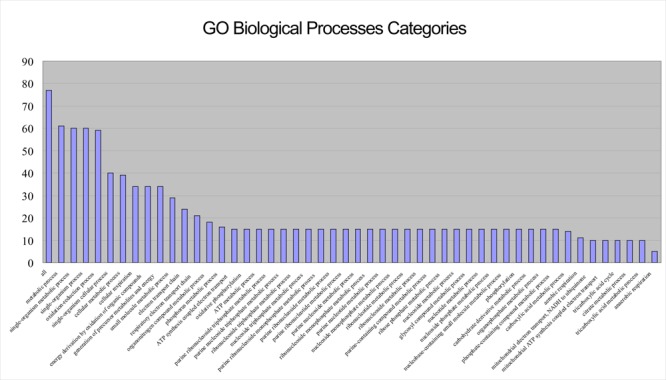
**Bar chart showing the GO biological process categories for the 77 proteins (or their encoding genes) in the top three shells of the *S. oneidensis* MR-1 electron transfer network (*p* < 0.0001).** The most representative category is “metabolic processes”.

As **Figure 2** shows, the proteins in the top three shells (with *k*_s_ 14, 13, and 10, respectively) are mainly located in the cytoplasm and inner membrane, with a small number in the periplasm, without outer membrane and extracellular space. Because there are dense interactions among network core proteins; by taking a larger fraction of their surface involved in many interactions, these proteins tend to be constrained, without further need of adaptive evolution that preferentially occurs in outer membrane and extracellular space (Kim et al., 2007). The Gene Ontology (GO) biological processes were exploited to obtain biological insights into these proteins; we found that the most representative category is metabolic processes (**Figure 3**). The results indicate that these proteins are mostly capable of cellular metabolism.

Then, KEGG enrichment analysis was used to probe into the details (**Table 1**). The enrichments results were highly consistent with the metabolism of *Shewanella* species. Metabolic pathways and carbon metabolism were the most common enrichments. The enrichment of oxidative phosphorylation reflected that it is the primary ATP synthesis pathway in *Shewanellae* (Venkateswaran et al., 1999). Enrichments of glyoxylate and dicarboxylate metabolism (a variation of the TCA cycle) and the citrate cycle (TCA cycle) correspond to *Shewanella* species having a complete TCA cycle under aerobic conditions (Pinchuk et al., 2010), and pyruvate fermentation in *S. oneidensis* MR-1 can provide essential energy for cell survival (Pinchuk et al., 2011).

**Table 1:** KEGG enrichment analysis for the 77 proteins (or genes) in the top three shells of the *S. oneidensis* MR-1 electron transfer network (*p* < 0.0001).

KEGG Pathway	Number	*P*-value
Metabolic pathways	44	1.74E-20
Oxidative phosphorylation	28	5.69E-39
Carbon metabolism	24	1.30E-22
Microbial metabolism in diverse environments	24	1.11E-16
Methane metabolism	12	2.86E-14
Glyoxylate and dicarboxylate metabolism	12	1.12E-13
Two-component system	12	8.90E-07
Pyruvate metabolism	8	5.27E-08
Citrate cycle (TCA cycle)	6	5.96E-07

Methane metabolism was also identified as an important enriched pathway. This was because anaerobic methane oxidation can be carried out for interspecies electron transfer (Stams and Plugge, 2009). The processes are thought to help bacteria sustain growth in syntrophic communities, which differ markedly from pure cultures and occur where diverse microbes exist in natural environments (Rotaru et al., 2015; Smith et al., 2015). Furthermore, there were 24 proteins identified to associate with microbial metabolism in diverse environments, such as FccA (fumarate reductase), Mdh (malate dehydrogenase), and SdhABC (succinate dehydrogenase). These diverse metabolic capabilities imply that *S. oneidensis* MR-1 has evolved flexible metabolic mechanisms to survive in a wide variety of environmental conditions, which agrees with the observation that a wide variety of type and concentration of substrates (e.g., fumarate, malate, and succinate) can be utilized by *S. oneidensis* MR-1 (Pinchuk et al., 2010). More importantly, with such a variety of metabolic capabilities, *S. oneidensis* MR-1 can oxidize many different substrates in different environments. Then, the generated electrons can be delivered into the quinone pool by NADH-quinone reductase (Nqr) and NADH-ubiquinone oxidoreductase (Nuo), which are found in the top three shells.

### The Fourth Shell Is Responsible for Extracellular Electron Transfer

Unlike the top three shells, which contain no outer membrane or extracellular proteins, the fourth shell (*k*_s_ = 9) contains such proteins (**Figure 2**). We considered detailed subcellular localization information for all proteins in this shell (**Table 2**). The outer membrane proteins (DmsF, MtrB, MtrE, SO_1659, SO_4359) and extracellular proteins (DmsA, DmsB, MtrC, MtrF, OmcA, SO_4357, SO_4358) in this shell are mostly functionally important for EET, as indicated by previous studies (Coursolle and Gralnick, 2010, 2012). Furthermore, proteins in this shell are broadly located in all five compartments of *S. oneidensis* MR-1 (i.e., the cytoplasm, inner membrane, periplasm, outer membrane and extracellular). Such diversified subcellular localization of proteins endows *S. oneidensis* MR-1 with the important EET ability, since electrons must be transferred from cytoplasm, via inner membrane, periplasm and outer membrane, to extracellular electron acceptors.

**Table 2:** Subcellular localization of proteins in the fourth shell of the *S. oneidensis* MR-1 electron transfer network.

Subcellular	Numbers	Proteins
Cy	6	AceE, LpdA, NapF, RnfB, SO_4504, TorD
IM	19	CcmA, CcmB, CcmC, CcmE, CcmF, CcmG, CcmH, CcmI, CoxB, CoxC, CymA, NapH, Ndh, NrfA, RnfD, RnfE, SirE, SirF, TorC
Pe	16	DmsE, DmsG, FdhX-2, MtrA, MtrD, NapA, NapB, NapD, NapG, PhsA, RnfC, RnfG, SO_4620, SO_4360, SO_4362, TorA
OM	5	DmsF, MtrB, MtrE, SO_1659, SO_4359
Ex	7	DmsA, DmsB, MtrC, MtrF, OmcA, SO_4357, SO_4358

*Ex, extracellular; OM, outer membrane; Pe, periplasm; IM, inner membrane; Cy, cytoplasm.*

**FIGURE 4:**
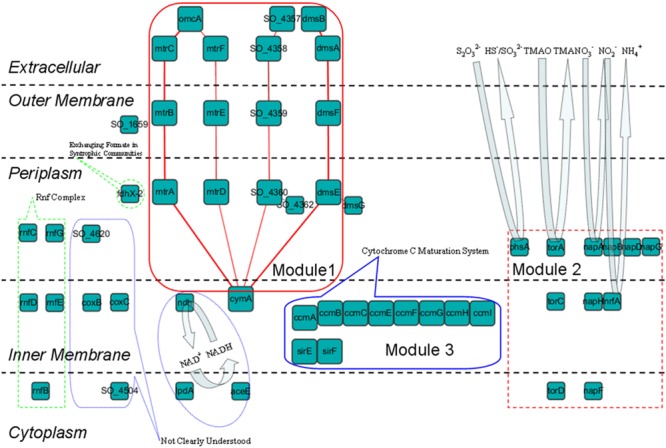
**Subcellular localization and functional roles of the proteins in the fourth shell of the *S. oneidensis* MR-1 electron transfer network are illustrated schematically (protein interactions are elided).** Module 1: reduction of extracellular insoluble electron acceptors, four EET pathways are marked by red lines; Module 2: reduction of extracellular soluble electron acceptors; Module 3: cytochrome *c* maturation (Ccm) system. The other proteins are also clustered and labeled with their functions.

We found that the proteins in this shell can be categorized into several modules according to their roles in EET (**Figure 4**):

#### Module 1 – Reduction of Extracellular Insoluble Electron Acceptors

With these outer membrane and extracellular proteins previously mentioned in this section, *S. oneidensis* MR-1 is capable of transferring electrons generated in the cytoplasm and gathered in the quinone pool (see The Top Three Shells Take Charge of Electron Generation) to extracellular insoluble acceptors. According to reported literatures (Coursolle and Gralnick, 2010, 2012), four EET pathways are formed from these proteins and their functional partners. These pathways include: (1) the MtrCAB pathway: CymA → MtrA → MtrB → MtrC → OmcA (Shi et al., 2012), and (2) the MtrDEF pathway: CymA → MtrD → MtrE → MtrF → OmcA (Breuer et al., 2012, 2014). In these two pathways, inner membrane CymA obtains electrons from the quinone pool, then transfers them via periplasmic MtrA/MtrD, outer membrane MtrB/MtrE, and extracellular MtrC-OmcA/MtrF-OmcA complexes, respectively, and finally to extracellular electron acceptors. These two pathways are considered to be metal reduction pathways. (3) The DMSO pathway: CymA → DmsE → DmsF → DmsAB complex; it was found that DmsE and DmsF play important roles in DMSO reduction, and the DMSO pathway was thus proposed (Gralnick et al., 2006). (4) The SO_4360-57 pathway: CymA → SO_4360 → SO_4359 → SO_4358/4357; analysis of homologs and subcellular localization has revealed that SO_4360/4359 are similar to MtrAB/MtrDE/DmsEF (Ding et al., 2014). That is, this pathway might be functionally redundant to the other three EET pathways. These results are consistent with Coursolle and Gralnick (2010, 2012) work, in which they deduced that the pathway shared overlapping functionality with the DMSO and MtrDEF pathways.

#### Module 2 – Reduction of Extracellular Soluble Electron Acceptors

This module deals with extracellular soluble electron acceptors that can be respired inside the cell. The *napDAGHB* gene cluster encodes nitrate reductase (NapA) and accessory proteins, the *nrfA* gene encodes the nitrite reductase (NrfA); they reduce nitrate to ammonium in a two-step manner in *S. oneidensis* MR-1, that is, reduction of nitrate to nitrite by NapA and followed by reduction of nitrite to ammonium by NrfA (Gao et al., 2009). The *torCAD* genes are three conserved structural components of the trimethylamine *N*-oxide (TMAO) respiratory system, which encode the Tor pathway that endows *Shewanella* species to use TMAO as a terminal electron acceptor for extracellular anaerobic respiration (Gon et al., 2002). PhsA is also functionally important for reduction of extracellular soluble electron acceptors, since Burns and DiChristina have shown that the anaerobic respiration of elemental sulfur and thiosulfate by *S. oneidensis* MR-1 requires PsrA, which is a homolog of PhsA (Burns and DiChristina, 2009).

#### Module 3 – Cytochrome *c* Maturation System

It has been shown that multiple post-translational modifications are required to synthesize the components of the EET pathways (such as the MtrCAB pathway). With reference to *c*-type cytochromes, this process is assured by the inner membrane proteins CcmABCEFGHI in this shell. These proteins are the components of the cytochrome *c* maturation (Ccm) system, which loads heme into the apocytochromes *c* to form mature cytochromes, such as MtrA and MtrC (Goldbeck et al., 2013). Be similar with the Ccm family proteins, the heme synthetases SirE and SirF in this shell also play an important role in the maturation of *c*-type cytochromes (Brockman, 2014).

#### Other Proteins Related to Extracellular Electron Transfer

The other proteins in the fourth shell have also been investigated. FdhX-2 is a formate dehydrogenase, gene expression of the *fdh* family genes has been shown to be significantly increased in syntrophic communities between *S. oneidensis* and *Escherichia coli* (Wang et al., 2015). The results of Wang et al. strongly suggest that the exchange of formate is favored in such a mutualistic condition, which might be because that formate serves as an electron carrier for direct interspecies EET between these two species (Shrestha and Rotaru, 2014). Despite no report in *Shewanella* species, the membrane-bound Rnf complex encoded by *rnfBCDEG* can combine carbon dioxide fixation with the generation and use of a sodium ion gradient for ATP synthesis in many bacteria, and this complex has been shown to be a major electron transport mechanism linked to energy conservation (Tremblay et al., 2012; Kracke et al., 2015).

Our results indicate that important *c*-type cytochromes were in the fourth shell of the network, including those that form the well-known EET pathways for reduction of extracellular insoluble electron acceptors and several respiratory systems for reduction of extracellular soluble electron acceptors. The accessory Ccm system and some other proteins linked to EET were also in this shell. From the network formation and extension view, that might be because many extracellular electron acceptors (such as various iron ores) exist in the environment of *S. oneidensis* MR-1, but the proteins in the top three shells cannot transfer electrons to the outside of cells. Thus, in order to take advantage of these extracellular electron acceptors, these EET-related *c*-type cytochromes were connected into the network. Then, other *c*-type cytochromes were continuously connected into the network to aid more efficient EET or accommodate other environmental conditions. In the long-term formation and extension of the network, these early *c*-type cytochromes that can transfer electrons outside of the cell gradually became part of a relative inner-shell of the network (here, the fourth).

### The *C*-type Cytochromes in the Remaining Shells Are Involved in Aiding Extracellular Electron Transfer

There is a huge periplasmic space between the inner membrane and outer membrane in *S. oneidensis* MR-1, and a 23.5 ± 3.7 nm distance has been determined by cryo-TEM measurements (Dohnalkova et al., 2011). To facilitate electrons crossing the periplasmic space of *S. oneidensis* MR-1, some periplasmic *c*-type cytochromes are needed.

Periplasmic tetraheme cytochrome *c* CctA can interact with its redox partners (CymA and MtrA) through a single heme. Therefore, it can serve as periplasmic electron relay to facilitate electrons transfer through the periplasmic space, that is, CymA → CctA → MtrA (Fonseca et al., 2013); CctA was found in shell 6 of the electron transfer network. As one of the most abundant periplasmic *c*-type cytochromes, ScyA in shell 7 has been shown to function as a mediator of electron transfer between CymA and CcpA (*c*-type cytochrome peroxidase) (Schutz et al., 2011; Fonseca et al., 2013). Furthermore, the cytochrome *bc*_1_ complex (encoded by the *pet* gene cluster) has been predicted to be the dominant electron donor to the *cbb*_3_-HCO-type oxidase (encoded by the *cco* gene cluster), and it has been shown that ScyA increases the electron flow from the *bc*_1_ complex to cytochrome *cbb*_3_-HCO oxidase (Yin et al., 2015). The expression level of the monoheme cytochrome *c* SorB and the decaheme cytochrome *c* SO_4360 were found to be upregulated with soluble iron(III) and oxygen as electron acceptors (Rosenbaum et al., 2012), and these two *c*-type cytochromes directly interact with each other, which raised a speculation that SorB can be used to help electrons reach SO_4360 and assist the SO_4360-57 pathway. It should be noted that fumarate reductase (FccA) also plays such an assistant role, just be similar with CctA (Fonseca et al., 2013). However, to support cellular metabolism as previous determined, this *c*-type cytochrome was found in the top three shells (see The Top Three Shells Take Charge of Electron Generation), rather than in a peripheral shell here.

Furthermore, although the physiological role remains to be examined *in vivo*, the octaheme tetrathionate reductase Otr displays nitrite, hydroxylamine, and tetrathionate reduction activities *in vitro* (Atkinson et al., 2007), which enhanced periplasmic electron transfer. It was also found that periplasmic *c*-type cytochromes can interact with several non *c*-type cytochrome proteins (**Supplementary Data Sheet S2**), which suggested that they also cooperated with non *c*-type cytochromes to facilitate periplasmic electron transfer in various environmental conditions. This mechanism offers one of the ways that ensure electricigens thrive in extreme environments. For example, Embree et al. (2014) recently reported that the expression of almost all *c*-type cytochromes of *G. sulfurreducens* sharply decreased when the iron ion concentration decreased, but the expression of *c*-type cytochrome GSU3274 increased gradually in these conditions, which can be reasonably interpreted to mean that GSU3274 is used in electron transfer by interacting with other non *c*-type cytochrome proteins, when the iron concentration becomes extreme limited.

Therefore, overall, functioning as multiple electron mediators or enhancing periplasmic electron transfer, the *c*-type cytochromes in the peripheral shells of the *S. oneidensis* MR-1 electron transfer network can help electrons cross the periplasmic space and hence they are involved in aiding EET processes. Furthermore, it is also interested that most (18 in 24) of the *c*-type cytochromes in the shells with *k*_s_ values less than 9 are located in the periplasm (**Table 3**). We speculated that they will form some short-range channels with other proteins by transient protein interactions, such as those formed by CctA/FccA (CymA → CctA/FccA → MtrA) (Fonseca et al., 2013).

**Table 3:** Subcellular localization of *c*-type cytochromes in the periphery of the *S. oneidensis* MR-1 electron transfer network (*k*_s_ < 9).

K-shell	Cy//IM	Pe	OM//Ex
8	CcoO, CcoP		
7		CytcB, Otr, ScyA, SirA, SO_0717, SO_1413, SO_4047, SO_4048	SO_0939, SO_2930, SO_2931
6		CctA, SO_3056, SorB	
5		CcpA, Dhc, Shp, SO_3300, SO_3420	
4		SO_0714	
3	SO_4572		
1		SO_4142	

*Ex, extracellular; OM, outer membrane; Pe, periplasm; IM, inner membrane; Cy, cytoplasm.*

**FIGURE 5:**
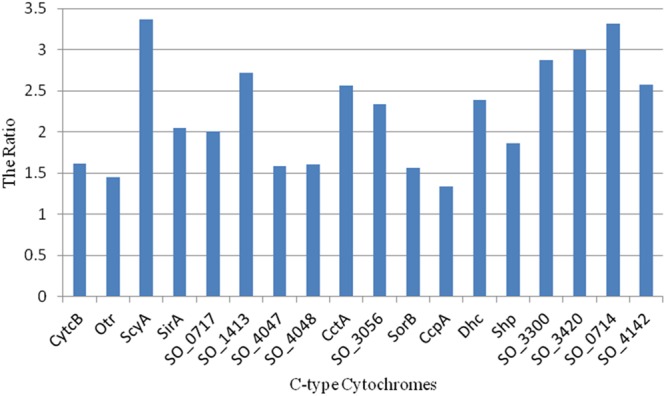
**Ratio of *DR_100* of the 18 periplasmic *c*-type cytochromes in the periphery of the *S. oneidensis* MR-1 electron transfer network (*k*_s_ < 9) to the average *DR_100* of the other proteins in the corresponding shells**.

To assess this, we analyzed the protein disordered regions in these 18 periplasmic *c*-type cytochromes and computed their *DR_100*. All of the 18 *c*-type cytochromes had a high level of disordered content compared with the average *DR_100* of the other proteins in the corresponding shells (**Figure 5**). Although protein disordered regions can fluctuate rapidly through a range of conformations, such conformational flexibility of disordered protein regions are quickly lost upon binding, which will reduce the overall free energy of binding and lead to weaker and more transient interactions (van der Lee et al., 2014).

**FIGURE 6:**
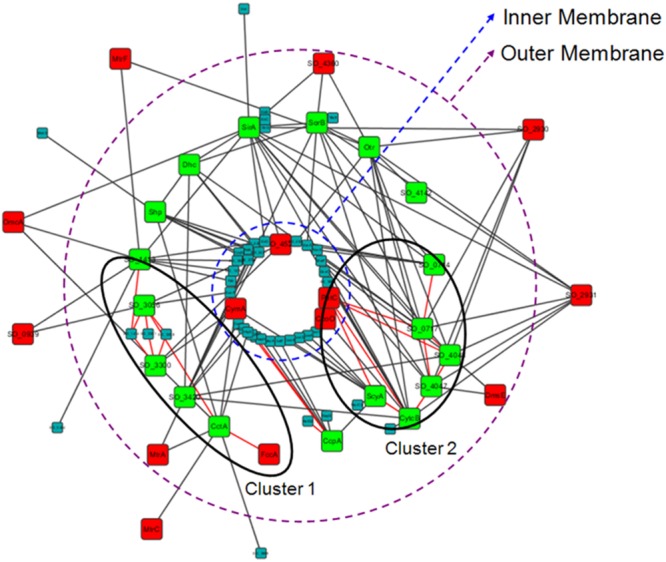
**Highly interconnected interactions of the 18 periplasmic *c*-type cytochromes in the periphery of the *S. oneidensis* MR-1 electron transfer network (*k*_s_ < 9).** These *c*-type cytochromes are indicated by green nodes, and their interacted partners are indicated by red nodes (other *c*-type cytochromes) or small nodes (non *c*-type cytochromes), respectively. Domain-domain interactions are shown with red lines, indicating that two clusters are formed.

We then analyzed the PPIs of these 18 periplasmic *c*-type cytochromes with all of their direct protein partners (interactions among these partners were not considered). As **Figure 6** shows, these *c*-type cytochromes (green nodes) were highly interconnected. Indeed, the density of this sub-network was 2.12-times higher than that of the whole electron transfer network, even though many interactions were not considered here. Their interaction partners included both other *c*-type cytochromes (red nodes in **Figure 6**) and non *c*-type cytochromes (**Figure 6**, small nodes). To further investigate whether weaker interactions could be formed, we performed domain-domain interaction (DDI, which are correspond to strong interaction) analysis for this sub-network (see **Supplementary Data Sheet S6** for protein domain). We found that there are only a few DDIs in the sub-network (**Figure 6**, red lines), and therefore, most of these periplasmic *c*-type cytochromes make weak, transient interactions with other proteins, rather than permanent interactions. Then, a dynamic electron transfer network forms in periplasm via the high frequency of transient protein interactions, as discussed elsewhere (Sturm et al., 2015). Since periplasmic electron transfer processes involve in assigning specific *c*-type cytochromes for particular electron acceptors and triggering of different pathways to achieve electron transfer (Sturm et al., 2015), thus the assignment of these periplasmic *c*-type cytochromes to different parts of the network (different shells here, see **Table 3**) is an effective strategy to achieve fast and efficient periplasmic electron transfer.

Furthermore, although there were only a few DDIs in this sub-network, two clusters were clearly formed from these DDIs (**Figure 6**). Previous studies have concluded that DDIs correspond to strong interactions that form functional modules in PPI networks (Kim et al., 2014). We therefore suggest that two functional clusters exist in periplasmic electron transfer (**Figure 6**, clusters 1 and 2). These clusters could be used to assist electrons to reach MtrA and DmsE, respectively, completing the most representative EET pathways: the MtrCAB pathway and the DMSO pathway. In addition to these EET pathways, they can also aid other proteins located at outer membrane or extracellularly (**Figure 6**).

## Conclusion

*Shewanella oneidensis* MR-1 is able to utilize a wide variety of extracellular solid electron acceptors such as iron or manganese oxides, which implies that it has evolved effective EET strategies (Kasai et al., 2015). Typically, this species uses numerous diverse biological pathways to efficiently perform such processes, which means that there is an interconnected network existent. As networks have been shown to strongly correlate with their function, and previous studies have shown high efficiency in the prediction of biological relevancy using network topology (Planas-Iglesias et al., 2012; Huang et al., 2013; Mukhopadhyay and Maulik, 2014), we thus explored such EET processes through an electron transfer network in *S. oneidensis* MR-1. We identified that protein disordered regions played an important role during the formation and extension of the electron transfer network, by analyzing the average disordered regions of proteins in every shell of the network. We also found that there are distinct functional parts in the network, and the functional significance of the various shells was discussed. Such a network-based study can be helpful for understanding potential EET processes in *S. oneidensis* MR-1.

## Author Contributions

DD designed the study, carried out the study and drafted the manuscript; LL helped construct the network and write the manuscript; CS helped write the manuscript; XS conceived of the study and was the lead writer of the manuscript. All authors read and approved the final manuscript.

## Conflict of Interest Statement

The authors declare that the research was conducted in the absence of any commercial or financial relationships that could be construed as a potential conflict of interest.

## Footnotes

1 http://www.genome.jp/dbget-bin/get_linkdb?-t+genes+genome:T00099

2 http://string.embl.de

3 http://bioinfo.protres.ru/IsUnstruct/

4 http://www.psort.org/psortb

5 http://cello.life.nctu.edu.tw/

6 http://pfam.xfam.org/

7 http://ffas.sanfordburnham.org

8 http://3did.irbbarcelona.org/

## Supplementary Material

DATA SHEET S1 **Four hundred and eighty-one electron transfer proteins which were identified to be potentially involved in the EET processes**.

DATA SHEET S2 **Electron transfer network which was obtained from STRING (December 2014)**.

DATA SHEET S3 **K-shell values for the proteins in the final electron transfer network**.

DATA SHEET S4 **The numbers of disordered regions for the proteins (aa length ≥ 100) in the final electron transfer network**.

DATA SHEET S5 **Subcellular localization for the proteins in the final electron transfer network**.

DATA SHEET S6 **Protein domains for the proteins in the final electron transfer network**.

METHODS **Detailed information on the methods used**.
